# Endovascular Treatment of a Contained Rupture of a Penetrating Aortic Ulcer in a Young Patient

**DOI:** 10.7759/cureus.56428

**Published:** 2024-03-18

**Authors:** Alexander T Daskalov

**Affiliations:** 1 Vascular Surgery, Acibadem City Clinic Tokuda Hospital, Sofia, BGR

**Keywords:** penetrating aortic ulcer, aortic repair, stent-graft, endovascular, contained rupture

## Abstract

Penetrating aortic ulcer (PAU) is a component of acute aortic syndromes (AASs), encompassing a range of potentially life-threatening aortic conditions such as dissection, intramural hematoma (IMH), and PAU itself. Ruptured PAU constitutes an emergency requiring surgical intervention. Here, we present a case involving a 47-year-old male patient admitted to our emergency department due to severe abdominal pain, malaise, and tenderness of the abdominal wall, which commenced abruptly several hours prior. An emergency CT scan revealed a large pseudoaneurysm of the infrarenal abdominal aorta, which was found with moderate atherosclerosis and no evidence of other dilated or aneurysmal segments. The patient underwent successful endovascular treatment and was discharged four days later without complications. Follow-up examination after two months demonstrated a patent graft and reduction of the aneurysmal sac.

## Introduction

A penetrating aortic/atherosclerotic ulcer (PAU) is characterized by a breach in the inner layer of the aorta due to atherosclerotic disease. On radiological imaging, it appears as a localized bulge of contrast material extending beyond the inner layer [1]. Severe damage to the aortic wall can lead to the formation of an aneurysm, resulting in a saccular pseudoaneurysm [2]. An aneurysmal PAU that is symptomatic and extensive is thought to pose an elevated risk of rupture, although the precise likelihood of rupture remains uncertain. Estimates indicate that approximately 14%-40% of patients either present with or progress to rupture [3]. PAUs are more commonly observed in elderly patients with severe atherosclerosis. While they can be identified in all segments of the aorta, they are particularly prevalent in the thoracic region, with the descending thoracic aorta being the most common site (62%) [4]. The rupture rate for PAU in acute presentations has been reported as high as 38%, significantly surpassing that observed for aortic dissection [5]. Indications for repair include persistent or recurrent symptoms, rupture, or the development of a pseudoaneurysm [1]. Surgical treatment is recommended for cases of PAU complicated by aneurysm expansion, regardless of size, rupture, peripheral embolization, or uncontrolled pain [1]. Open surgical repair with graft interposition is associated with high operative morbidity and mortality [6]. Endovascular treatment offers a less invasive and appealing alternative, as it can be conducted under local anesthesia, making it suitable for high-risk patients and potentially decreasing complications [7].

## Case presentation

**Figure 1:**
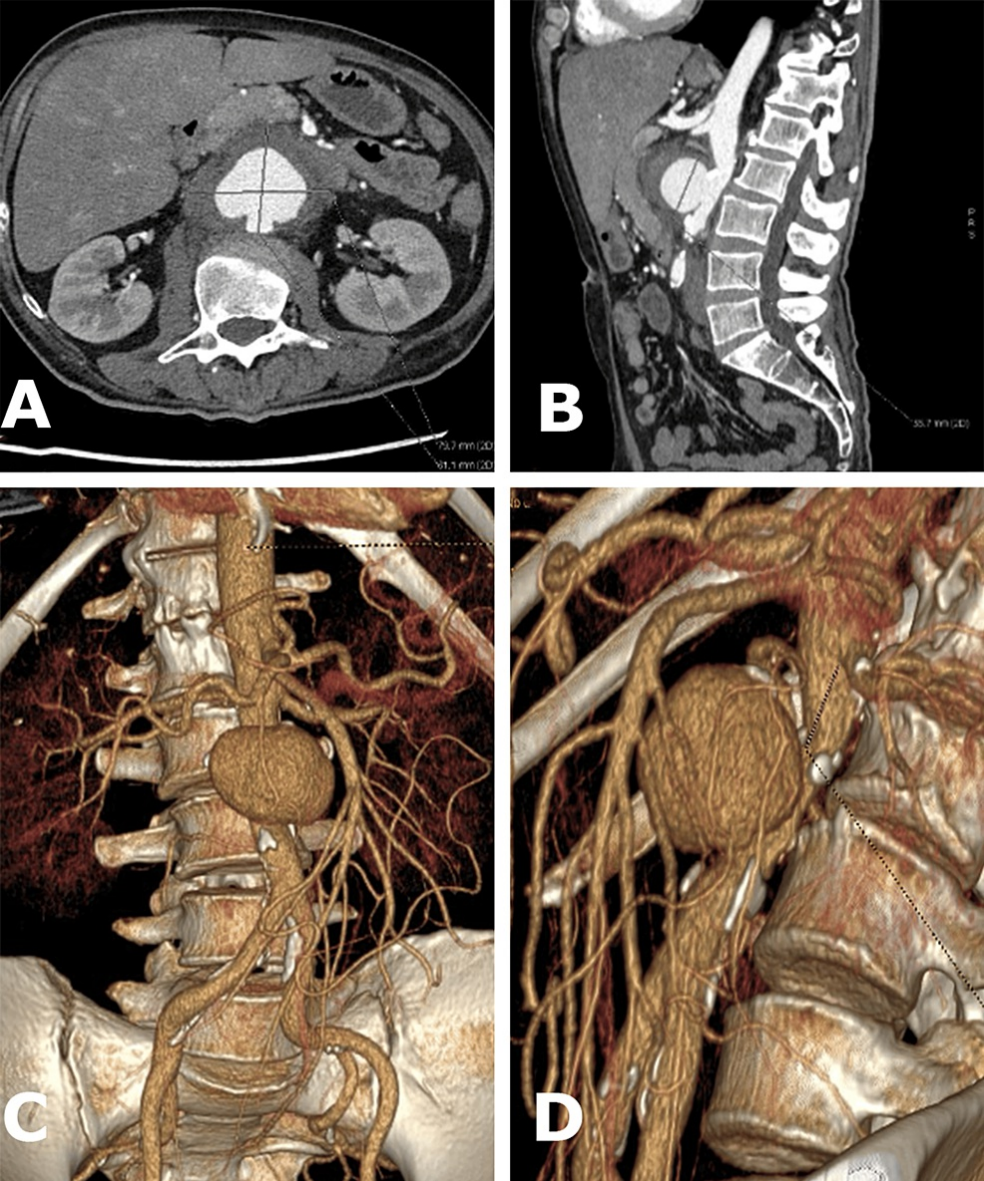
(A)-(D) Saccular pseudoaneurysm with the contained rupture of the infrarenal aorta.

A 47-year-old man presented at the emergency department in distress, reporting severe abdominal pain with abrupt onset several hours earlier. Upon initial examination, he exhibited abdominal tenderness, exacerbated by palpation, but no signs of rigidity were noted. All peripheral pulsations were intact. He was slightly hypotensive at 100/70 mmHg, and he displayed tachycardia, with a heart rate of 120 beats per minute. There were no reported comorbidities, and no other abnormal physical findings were observed. Laboratory tests revealed moderate anemia (hemoglobin 85 g/L), a slightly elevated white blood cell count (12.49 x 10^9^), a platelet count within the normal range (118 x 10^9^), a normal creatinine level (66.3 umol/L), and a moderately elevated C-reactive protein (29.95 mg/L). The patient was not febrile. A habitual smoker, he had no history of previous abdominal discomfort or traumatic events. An urgent contrast-enhanced CT scan was performed that revealed a sizable saccular aneurysm measuring 8 cm x 6 cm x 6 cm in the infrarenal aorta, suspected to be complicated by a contained rupture. Notably, there were no signs of extensive atherosclerosis or additional aneurysmal segments involving the aorta or its branches (Figure 1).

**Figure 2:**
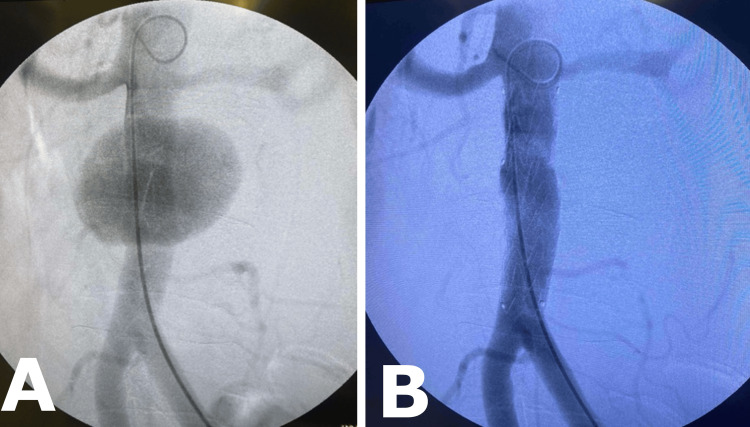
(A) and (B) Initial aortography and final result with no signs of endoleak.

Due to the favorable positioning of the aneurysmal neck and the overall health of the surrounding aorta, along with the potential for further evolution of the contained rupture, we chose an endovascular approach. The proximal aneurysm neck began approximately 27 mm below the renal arteries, with an aperture width of 20 mm, and a distal segment extending to the aortic bifurcation providing a seal zone of approximately 37 mm. However, the aortic neck at the level of the renal arteries measured around 16-17 mm, which was slightly too narrow for the smallest available tube graft. Considering another option, a complete bifurcated system was deemed unattractive due to the short length of the infrarenal aorta (85 mm), the too-narrow aortic bifurcation of about 12 mm, and the risk of compromising the opening of the contralateral limb. Considering these concerns, we opted to utilize an Iliac Extension Stent Graft (20/20/82 Medtronic, Minneapolis, MN), readily available off-the-shelf, albeit off-label for this purpose. Using a trans-femoral approach from the right side, an aortography was performed, and the iliac extension was accurately placed. The final arteriogram revealed a perfect result with no signs of endoleak (Figure 2).

The patient was discharged on the third postoperative day without any complications. A follow-up examination two months after the procedure revealed a patent stent graft and a slight reduction in the aneurysmal diameter.

## Discussion

Originally identified as a distinctive condition in 1986 by Stanson et al., an isolated PAU is characterized by the disruption of the arterial intima and elastic lamina, extending into the media within the atherosclerotic aorta. This disruption leads to a localized bulging through an area of vascular calcification with accompanying arterial flow [1]. PAU is often discovered incidentally, fully asymptomatic. While an isolated PAU may follow a benign clinical course, such as in cases of asymptomatic, stable ulceration incidentally observed, it also has the potential to grow or develop an associated intramural hematoma (IMH), dissection, pseudoaneurysm, or rupture [1-4].

PAU tends to occur more frequently in elderly patients who have advanced and severe systemic atherosclerotic disease. Consequently, it is often associated with serious health issues such as hypertension, coronary artery disease, other cardiac risk factors, and occlusive diseases in the carotid or peripheral arteries [4]. However, in the current case, we encountered a discrepancy concerning these predisposing factors, considering the patient's age, the absence of severe atherosclerosis, and the exact location of the pseudoaneurysm.

Reviewing the literature suggests mainly three potential differential diagnoses for such findings: abdominal aortic pseudoaneurysm (AAP), mycotic aneurysm, and complicated PAU. AAP is an uncommon and serious consequence of aortic damage, typically seen following penetrating injuries or, less frequently, blunt trauma, often associated with vehicular accidents. In existing literature, the duration between the initial injury and AAP diagnosis varies considerably, spanning from days to even years. The precise cause for this discrepancy remains uncertain; however, it could be theorized that penetrating injuries with high velocity, exerting substantial force on the tissue, might induce subtle localized damage to the vessel wall, which becomes apparent during the fibrotic healing process later on [8].

In the present case, the patient had no history of trauma, making traumatic etiology unlikely. Another possible diagnosis was a mycotic aneurysm. The term *mycotic aneurysm* was first coined by William Osler in 1885. However, it is misleading since the majority of mycotic aneurysms are caused by bacterial infections rather than fungal ones. The classic triad of fever, abdominal pain, and pulsatile abdominal mass is commonly associated with this condition, yet it may not always manifest, posing a challenge in diagnosing a mycotic aneurysm and necessitating a high level of suspicion. The natural history of a mycotic abdominal aortic aneurysm (AAA) typically involves rapid progression to rupture and often death [9].

In our case, the patient was afebrile, with only a slight elevation in white blood cell count, which normalized upon discharge. The C-reactive protein level was 29.95 mg/L but decreased during hospitalization and normalized two months after discharge. This led us to consider *ruptured PAU* as the most probable cause of our emergency case, particularly considering the sudden onset of symptoms, the presence of anemia, thrombocytopenia, and hypotension.

PAUs are more commonly found in the descending aorta [10] and are rarely observed in the abdominal segment, especially in younger patients. PAUs involving the descending thoracic aorta require urgent management in symptomatic patients with persistent or increasing pain, hemodynamic instability, those with expanding IMH, intrabronchial pressure syndrome (IBPS), and/or hemothorax, as well as those presenting with hemopericardium. Emergency treatment is also necessary in cases with a larger aortic diameter (>60 mm), saccular morphology of associated aneurysm, imminent or evident signs of rupture, and fistulous communication with the airway or esophagus [5]. The emergency intervention was warranted in our case due to the sudden onset of symptoms, the maximal diameter of the aneurysm being approximately 80 mm, and the indication of contained rupture with the potential for progression to life-threatening abdominal hemorrhage.

Possible treatment options for aortic pseudoaneurysms include an open surgical repair, endovascular repair, pseudoaneurysmal sac thrombosis induction through direct thrombin injection [11], and coil embolization [12]. The decision regarding whether to opt for an open or endovascular method relies on factors such as the patient's age, any accompanying health conditions, technical considerations such as the shape of the lesion and variations in anatomy, the types of prostheses available, and the expertise of medical personnel and centers. Open surgical repair typically involves resection of the pseudoaneurysm with an interposing graft or repairing the aorta using a lateral Dacron patch aortoplasty, as outlined by Pisters et al. [13].

Endovascular repair has emerged as a treatment option for AAP, utilizing stent grafts and balloon-expandable bifurcated endoprostheses [14,15]. Bechara-Zamudio et al. documented a case involving a 22-year-old male who presented with significant intra-abdominal hemorrhage. Diagnosis of AAP via CT scan led to treatment with a balloon-expandable bifurcated endoprosthesis.

Despite the widespread acceptance of endovascular treatment for AAA using dedicated aortic endograft systems, their applicability in cases of abdominal PAUs or AAP can be limited, often due to aortic diameters being within the normal or even narrower range. In such scenarios where the placement of a bifurcated self-expanding aortic graft is not feasible, aortomonoiliac reconstruction, as reported by Hinchliffe et al. [16], could be considered; however, this approach typically necessitates subsequent cross-over bypass and, in many cases, embolization of the contralateral iliac artery.

An alternative option for situations involving a small diameter of the aortic bifurcation is the utilization of an AFX stent graft. Manufactured by Endologix in Irvine, CA, the AFX stent graft is specifically engineered to address the challenge posed by narrow aortic bifurcations. Unlike traditional bifurcated endografts, which may subject iliac limbs to potential competition within the restricted lumen, thereby heightening the risk of stenosis or thrombosis, the AFX stent graft is designed for direct attachment onto the aortic bifurcation. This design feature aims to mitigate limb competition within the distal aorta, offering particular advantages in cases with narrow aortic bifurcations.

Alternative approaches to conventional methods such as open surgical repair or endovascular repair have been reported in various studies. In a case described by Geckeis et al. [11], a 63-year-old man experienced the formation of a significant pseudoaneurysm in the abdominal aorta after undergoing surgical fenestration and patch aortoplasty to address acute type B aortic dissection with malperfusion syndrome. The authors successfully induced complete clotting within the pseudoaneurysm sac by administering 1,500 thrombin units via transcatheter delivery.

Salsamendi et al. [12] utilized endovascular coil embolization to manage a posterior aortic aneurysm situated slightly above the origin of the renal arteries, along with a smaller broad-based AAP adjacent to the right superior mesenteric artery. In this particular case, endovascular coil embolization was considered a feasible alternative for treating an AAP that was not suitable for stent grafting or traditional open surgical repair.

We chose an endovascular approach due to the urgent condition of the patient, the absence of suspicion of a mycotic aneurysm, and favorable anatomy regarding the location of the PAU. The relatively normal aortic morphology on both sides of the ulcer facilitated the acquisition of landing zones for better graft stability. According to the literature, a minimum length of 1.5-2 cm at each end of the landing zone is required [17]. Fortunately, we had sufficient proximal (27 mm) and distal (37 mm) landing zones of adequate length and quality. The only challenge we encountered was the narrow diameter of the entire abdominal aorta, particularly at the aortic bifurcation. Since we did not have an appropriate off-the-shelf aortic tube graft or a dedicated bifurcated system to address the narrow aorta (such as the AFX stent graft), we decided to use an off-label Iliac Extension Stent Graft, which resulted in a successful outcome.

## Conclusions

In this current case report, we present the successful treatment of a contained rupture of PAU in a young patient, a remarkably rare occurrence. Ruptured PAU represents a distinctive clinical entity, with ongoing debates surrounding the understanding of its natural history and optimal management strategies. This ambiguity is partly due to the infrequency of such cases. Treatment strategies are not firmly established, but it is commonly agreed upon that conservative management is preferred for small, non-aneurysmal, asymptomatic PAUs. In cases necessitating intervention, endovascular approaches offer a viable solution with favorable long-term outcomes, particularly in urgent scenarios. However, due to the rarity of these cases, dedicated endovascular solutions may not always be readily available. Comprehensive studies investigating the treatment strategies for PAU are warranted to guide clinicians in determining the most appropriate course of action.
